# Update of the sequential organ failure assessment score: current status and challenges?

**DOI:** 10.3389/fmed.2025.1733090

**Published:** 2026-01-13

**Authors:** Jiafei Yu, Kangwei Sun, Yiping Zhou, Yushi Fan, Xinyun Zhang, Heyu Chen, Lanxin Cao, Kai Zhang, Gensheng Zhang

**Affiliations:** 1 Zhejiang Chinese Medical University, Hangzhou, China; 2 Department of Critical Care Medicine, Haiyan People's Hospital, Zhejiang, China; 3 Department of Critical Care Medicine, Second Affiliated Hospital, Zhejiang University School of Medicine, Hangzhou, China; 4 Department of Emergency Medicine, Dongyang People' Hospital of Wenzhou Medical University, Zhejiang, China; 5 Department of Critical Care Medicine, Taizhou Hospital of Zhejiang Province, Zhejiang, China; 6 Department of Critical Care Medicine, Zhejiang Cancer Hospital, Hangzhou, China; 7 Key Laboratory of Multiple Organ Failure (Zhejiang University), Ministry of Education, Hangzhou, China

**Keywords:** deficiencies, intensive care unit, modifications, multiple organ dysfunction, SOFA score

## Abstract

The sequential organ failure assessment (SOFA) score has been widely used for approximately 30 years for the clinical assessment and monitoring of organ dysfunction in patients. However, advancements in medical science and clinical practice have identified several limitations of the SOFA score, underscoring the need for its revision. This paper synthesizes and summarizes the recent advances and provides a comprehensive understanding of the deficiencies of the SOFA score, which requires certain refinements. Furthermore, this work presents a scientific basis and direction for further modifications to enhance the clinical utility of the score.

## Introduction

To quantify the extent of organ dysfunction in individual patients, scholars from the European Society of Intensive Care Medicine (ESICM) developed the straightforward sequential organ failure assessment (SOFA) score during a consensus meeting of the Working Group on Sepsis-Related Problems at the European Annual Congress of Intensive Care Medicine in Versailles in 1994; the final version of the score was published in 1996 (1). The SOFA score comprises six organ systems, including respiratory, coagulation, hepatic, cardiovascular, central nervous system (CNS), and renal, providing a comprehensive reflection of their functional status. Each system is assigned a score between 0 and 4, with higher scores indicating a more severe impairment of the organ function. The SOFA score offers two key advantages: (1) permits the continuous monitoring of an organ-system function on a daily basis, which is particularly beneficial considering critical care (2); (2) demonstrates strong associations with important clinical outcomes, including in-hospital mortality (3), length of hospitalization (4), and the risk of acquiring infections in the intensive care unit (ICU) (5). Consequently, the SOFA score is highly valuable in clinical decision-making, disease management, and research.

In “Sepsis 3.0,” the SOFA score was identified as a measure to determine the severity of disease and as a tool for mortality risk-stratification (6). However, its limitations are being revealed considering the emerging pharmacological therapies, experimental studies, and advanced organ support modalities. First, the SOFA score has certain components that require laboratory assays, such as obtaining a blood gas analysis, platelet, bilirubin, and blood creatinine levels, which cannot be rapidly and repeatedly performed in a feasible manner in clinical practice, thereby weakening its feasibility. Second, the score does not consider these updates in medications and organ support modalities, which may impact its predictive efficacy. Third, the application of the SOFA score may vary depending on various clinical settings and patient populations, requiring further research (3). Therefore, revision of the SOFA score is being proposed to better adapt to the demands of contemporary medicine. In 2025, the SOFA-2 score was developed and validated using 10 international multicenter cohorts comprising 3.3 million adult ICU patients. The updated score now incorporates commonly used drugs and mechanical organ support modalities that were rarely or not used when the original version was published in 1996. Some thresholds were modified to improve predictive validity against ICU mortality (7). In this review, we analyzed and explored the current status and challenges of SOFA scoring, attempting to identify the potential strategies for its improvement.

## Development of the SOFA score

The SOFA score was initially utilized for the assessment of sepsis-related organ dysfunction following the 1994 consensus conference considering its development. Subsequently, it was widely recognized and validated for the assessment of severity in critical care (1). Scholars have recently implemented several variations to the original SOFA score to simplify it and to improve its accuracy (48, 49). The following components are included: Modified SOFA for Oncology Patients (mSOFA) (8), Modified Cardiovascular SOFA (mCV-SOFA) (9), Extra-renal SOFA (10), Chronic Liver Failure SOFA (CLIF-SOFA) (11), Mexican SOFA (Mex-SOFA) (12), and Pediatric SOFA (pSOFA) (13) (Table 1). However, these scores are specific to patient populations. For example, the mSOFA score has been used in patients with severe neurological injury to determine the degree of organ dysfunction; CLIF-SOFA is specifically designed to assess patients with liver failure. Regardless, the sensitivity and predictive ability of these scores are equal to or better than those of the original SOFA. The mCV-SOFA score improved the overall performance of the SOFA in predicting patient outcomes (9); CLIF-SOFA demonstrated a better performance in predicting the 28-day mortality (11); the Mex-SOFA score was comparable to that of the original SOFA in predicting the mortality in critically ill patients (12); the pSOFA score was demonstrated to be similar to or better than other pediatric organ dysfunction scores in terms of in-hospital mortality (13). Most importantly, when the SOFA score was modified, the score was more concise; however, the prognostic value was similar to or better than that of the original SOFA score.

**Table 1:** Type of modified sequential organ failure assessment score-based models.

**SOFA-based scoring systems**	**Respiratory**	**Coagulation**	**Hepatic**	**Cardiovascular**	**CNS**	**Renal**
Modified SOFA (mSOFA) (48)	Respiratory support not included	Platelet count	Bilirubin	Number of vasopressors; Blood pressure not included	/	Creatinine
Modified Cardiovascular SOFA (mCV-SOFA) (8)	PaO_2_/FiO_2_ ratio; Ventilator support consideration for values 3 and 4	Platelet count	Bilirubin	Lactate levels; Shock index; Number of vasopressors	GCS	Creatinine; Urinary output
Extra-renal SOFA (9)	PaO_2_/FiO_2_ rate; Ventilator support consideration for values 3 and 4	Platelet count	Bilirubin	MAP <70 mmHg; Usage of vasopressors/dobutamine	GCS	/
Chronic Liver Failure (CLIF-SOFA) (10)	PaO_2_/FiO_2_ ratio or SpO_2_/FiO_2_ ratio	Platelet count INR	Bilirubin	Use of vasopressors	Presence of grade II or IV hepatic encephalopathy	Creatinine; Renal replacement therapy
Mexican SOFA (Mex SOFA) (11)	SpO_2_/FiO_2_ ratio; Ventilator support consideration for values 3 and 4	Platelet count	/	MAP <70 mmHg; Use of vasopressors/ dobutamine	GCS	Creatinine; Urinary output
Pediatric SOFA (pSOFA) (12)	PaO_2_/FiO_2_ ratio or SpO_2_/FiO_2_ ratio; Ventilator support consideration for values 3 and 4	Platelet count	Bilirubin	MAP (age-adjusted); Vasopressors agents (age-adjusted)	GCS	Creatinine (age-adjusted)

GCS, Glasgow Coma Scale; INR, international normalized ratio; MAP, mean arterial pressure.

## Components and limitations of the original SOFA score

### Respiratory score

Arterial blood gases and invasive mechanical ventilation have been used as benchmarks for this part of the SOFA score, which not only increases patient discomfort, but may also increase the risk of puncture failure. In this regard, scholars have proposed using the SpO_2_/FiO_2_ ratio instead of the PaO_2_/FiO_2_ ratio and the measured SpO_2_ imputed by the conversion table to the PaO_2_ (Supplementary Table S1) (14). This substitution aims to reduce patient trauma, conserve healthcare resources, and improve the ease of assessment; this substitution has been found to be feasible (15). Regardless, its validity and accuracy need to be further validated and supported by large-sample clinical trials.

In addition, considering the rapid development of respiratory support techniques, the definition of “respiratory support” in the respiratory SOFA score has revealed its limitations. Traditional respiratory support mainly refers to invasive mechanical ventilation; however, the latest clinical practice has demonstrated that respiratory support is significantly more than that. Emerging modalities, such as high-flow nasal cannula (HFNC) oxygen therapy (16), non-invasive positive pressure ventilation (NIPPV), and veno-venous extracorporeal membrane oxygenation (VV-ECMO) (17), play an important role in the treatment of critically ill patients. Unfortunately, these support modalities have not been adequately reflected/adopted in the current SOFA scoring system, resulting in the relative inaccuracy or deficiency of the original SOFA score to predict the outcomes of patients requiring the aforementioned respiratory support.

Therefore, future research should focus on updating and expanding this portion of the SOFA score to better reflect the changes in clinical practice and the diversity of patients with various respiratory support techniques.

### Coagulation score

The platelet count has been used as a benchmark for this part of the SOFA score, and its changes are important in assessing the coagulation function and disease severity of patients. However, in addition to the progression of the disease, the platelet count is influenced by various other complex factors such as the medications (18), diet and nutritional status (19), which may lead to fluctuations in the platelet count and thus affect the accuracy of the SOFA score to a certain extent. In particular, platelet transfusion, which is a common clinical intervention, may directly alter the platelet count. However, the current SOFA scoring system does not explicitly consider platelet transfusion, which may lead to incorrect assessments of the organ-function recovery after treatment.

Scholars have proposed solutions to the aforementioned problem, such as suggesting that the daily score after platelet transfusion should be calculated from the lowest value before transfusion (20), reducing the immediate effect of platelet transfusion on the scoring results; however, this may simultaneously ignore the effect of treatment on the coagulation function of patients and the actual state of the overall condition, thus weakening the value of the SOFA score in evaluating the effectiveness of the treatment. On the other hand, studies have suggested that in disseminated intravascular coagulation associated with sepsis, elevated D-dimer tends to precede the abnormalities of other coagulation indices, suggesting its potential advantage in the early detection of coagulation disorders (21). Therefore, researchers have proposed the inclusion of D-dimer in the coagulation component of the SOFA score (21) to assess the coagulation status and prognostic risk of patients more comprehensively.

However, the lack of multi-center, large-sample randomized control studies limits our in-depth understanding and scientific evaluation of the clinical value of both. In the future, more high-quality clinical studies are expected to identify and incorporate indicators that may better predict the coagulation status, such as D-dimer and other SOFA blood fraction scores, to provide a more reliable basis for clinical decision-making.

### Cardiovascular score

This part of the SOFA score is based on blood pressure, the use of vasopressors (including epinephrine and norepinephrine), and positive inotropes (including dopamine and dobutamine) (9). The vasopressor of choice in early sepsis is dobutamine (22), which is used to assess the cardiovascular SOFA score. However, as clinical practice has significantly evolved, especially since 2008, when norepinephrine gradually replaced dobutamine as the first-line vasoactive drug (6), this change has led to a reduction in the proportion of cardiovascular SOFA scores of 2 points (23). Furthermore, even norepinephrine infusions well below 0.1 μg/(kg-min) for at least 1 h resulted in a SOFA score of 3 points for this component, consequently increasing it by 3 points only for this term of the cardiovascular proportion of the SOFA score. Recently, the increased use of metaraminol, terlipressin, and other adrenomimetic and vasoactive drugs in critically ill patients (24), as well as the application of veno-arterial extracorporeal membrane oxygenation (VA-ECMO), has further challenged the current SOFA scoring system.

In response to these changes, some studies have proposed the vasoactive inotropic score (VIS), a quantitative score for cardiovascular support medications (Supplementary Table S2), which standardizes different types of cardiovascular support medications by the dose conversion to facilitate comparability (25). Research has indicated that the SOFA score has improved its accuracy in predicting the 30-day mortality when the cardiovascular component of the SOFA score was replaced by the VIS score (26). However, whether the VIS score better reflects the cardiovascular component of the SOFA score needs further investigation.

In addition, as a marker of tissue hypoxia, lactate is considered as a simple, inexpensive, and sensitive tool for assessing organ dysfunction and for guiding fluid resuscitation in patients (27); it has been identified as an independent prognostic predictor in patients with sepsis (28). Recent studies have shown that the lactate concentration could be considered to quantify the severity of cardiovascular dysfunction via the SOFA score (28). This finding provides a new perspective on the cardiovascular component of SOFA scoring; however, more studies are required for clarity. Moreover, other tissue hypoxia factors, such as liver function and medications, may also increase lactate concentrations, which should be considered.

### Hepatic score

This part of the score was assessed using serum bilirubin levels, as hyperbilirubinemia is often secondary to sepsis-induced cholestasis, hemolysis, and direct hepatocellular injury. However, assessment of the liver function is the most frequently missing component of this scoring system (29), which may be because daily liver function monitoring is not routinely performed in clinical practice, particularly when the liver function of patients remains relatively normal, resulting in the absence of this critical information. In addition, reliance on bilirubin levels alone as an indicator of liver function has inherent limitations, as it does not adequately reflect the state of metabolism, detoxification, and integrated functions of the liver. Elevated bilirubin may also be due to hemolysis rather than liver dysfunction.

Researchers have proposed that the hepatic encephalopathy score should be used instead of bilirubin as the basis for this part of the assessment. However, hepatic encephalopathy is often observed in advanced stages of liver failure and may not provide an early warning for patients with early liver dysfunction. Whether there are potential indicators that reflect the liver function instead of bilirubin merits further investigations.

### CNS score

This section utilizes the Glasgow Coma Scale (GCS) as a benchmark; however, the GCS relies on the assessment of verbal function, which limits its use in intubated and/or sedated patients (30, 31). In addition, the subjective nature of the GCS may lead to scoring discrepancies. When the frequency of errors in the total SOFA score is greater than 1 or 2 points, it can be reportedly reduced by brief rater training, thereby reducing the inter-rater variability in SOFA scores for the same patient; however, its accuracy for CNS scoring was not adequately addressed in the study (32).

To overcome these limitations, the FOUR scale (Supplementary Table S3) and RASS scores were developed and used as alternatives to the modified version of the SOFA score. The FOUR scale not only assesses the ocular and motor responses, but also the brainstem reflexes and respiratory status, effectively compensating for the inadequacy of the GCS score in mechanically ventilated patients; this scale is recommended by the ESICM guidelines (33). However, the FOUR score omits the behavioral manifestations and psychometric features associated with the minimal impairment of consciousness, limiting its potential for a comprehensive assessment (34). Conversely, the RASS score is a standardized tool used to assess the level of consciousness and sedation in patients. It has gradually become an important tool for assessing the neurological function in the ICU due to its low inter-observer variability (35). Studies have shown that the RASS-based score is comparable to the traditional SOFA score in predicting the ICU mortality and in-hospital mortality among ICU patients (36, 37). However, there is no consensus regarding the optimal timing for patient assessment during sedation. Certain scholars have proposed that the assessment should occur 24 h after the cessation of sedative infusion (20), whereas others have suggested that the GCS value should remain consistent with the pre-tracheal intubation value throughout the entire period of sedation (1). Regardless, the clinical feasibility of these alternatives to the GCS score is frequently difficult to ascertain, and the accuracy and other aspects of their implementation must be further elucidated.

Considering the aforementioned limitations of the SOFA score with respect to the CNS component, a multicenter cross-sectional study revealed that the modified SOFA score excluding the CNS portion was more feasible and effective in predicting the ICU mortality rate than the original SOFA score in patients with severe sepsis (38). However, further clinical studies are evidently required to substantiate these results. Studies regarding the optimal index for assessing the CNS function remain limited, indicating a pivotal and challenging area for future research.

### Renal score

In this section, the blood creatinine levels and daily urine output are employed as the indicators for the renal score. However, in patients undergoing CRRT, the treatment has a significant effect on the blood creatinine values and urine output, consequently affecting the accuracy of the renal SOFA score.

Considering the aforementioned (20), modifying the renal SOFA scoring system to account for the direct impact of CRRT therapy on blood creatinine and urine output parameters is imperative.

Based on the aforementioned descriptions, the limitations and improvements of the original SOFA score are summarized in Table 2.

**Table 2:** Limitations and improvements of the original SOFA score.

**Organ/system**	**Current indicators**	**Limitations**	**Possible additions/alternatives measure**
Respiratory	PaO_2_/FiO_2_ ratio; ventilator support consideration for values 3 and 4	Puncture pain and puncture failure; Traditional respiratory support like invasive mechanical ventilation	SpO_2_/FiO_2_ ratio; HFNC; NIPPV; VV-ECMO
Coagulation	Platelet count	Platelet account affected by various factors like medications, diet, and nutritional status	D-dimer; platelet transfusion
Cardiovascular	MAP <70 mmHg; Usage of vasopressors/dobutamine	Only Adrenomimetic and vasoactive drugs evolved	Dobutamine, milrinone, levosimendan, antidiuretic hormone; lactate; VA-ECMO
Hepatic	Bilirubin	Missing frequently; incomprehensive	Hepatic encephalopathy score
CNS	GCS	Subjective; Relies on verbal function; Unavailable in intubated and/or sedated patients	Rater training; FOUR scale/RASS; GCS remain the pre-tracheal intubation value; GCS after sedation 24 h
Renal	Creatinine; urinary output	Impact on CRRT;	CRRT

CNS, central nervous system; CRRT, continuous renal replacement therapy; FOUR, Full Outline of Un-Responsiveness; GCS, Glasgow Coma Scale; HFNC, high-flow nasal cannula; NIPPV, non-invasive positive pressure ventilation; VV-ECMO, veno-venous extracorporeal membrane oxygenation; RASS, Richmond agitation-sedation score; VIS, vasoactive inotropic score.

### Other organs and indicators

Whether the original SOFA score fully reflects the function of all organs/systems, or simple and valid indicators that can reflect the function of other organs/systems should be included in the SOFA score, requires further consideration.

### Intestinal system

Intestinal tissues represent a crucial target organ for infectious injury, particularly considering septic shock. In such instances, visceral blood vessels undergo selective contraction, ensuring an adequate blood supply to the vital organs. This physiological response results in a range of complications, including gastrointestinal ischemia and hypoxia, epithelial necrosis and detachment, impaired intestinal barrier function, increased permeability, and the diffusion of bacteria, endotoxins, and other inflammatory mediators (39). These factors contribute to an excessive systemic inflammatory response and the subsequent dysfunction of other organs, amplifying patient suffering and hindering recovery. Patients with severe acute gastrointestinal injury (AGI) are also more likely to experience recurrent health issues and complications after discharge. This leads to higher readmission rates, puts additional strain on healthcare resources and creates substantial psychological and physiological burdens (40). Furthermore, the majority of patients receive antibiotic therapy, which often results in the depletion of beneficial intestinal bacteria, the proliferation of opportunistic pathogens, and intestinal flora dysbiosis and ectopia (41). Despite using short-term antibiotics, disturbances in the gut microbiome can persist for a long time (42), further complicating and worsening the patient condition. Therefore, inclusion of the intestinal system in the SOFA score must be urgently considered.

However, owing to the complexity of the intestinal function and lack of specific assessment indices, the original SOFA score has not included the intestinal system thus far (43). In 2012, the ESICM proposed the (AGIclassification system, which has been widely used for assessing the intestinal function in critically ill patients. A multicenter prospective study developed the gastrointestinal dysfunction score (GIDS; Supplementary Table S4) based on the AGI and integrated it with the SOFA score, which demonstrated to be superior in predicting the risk of mortality (44). Regardless, the table is overly complex and does not fully reflect all the intestinal functions (e.g., endocrine, immune, and battier).

Therefore, it is crucial to identify reliable indicators that can effectively reflect the function of the intestinal system. The recent increase in research regarding citrulline and the intestinal fatty acid binding protein (I-FABP) has resulted in significant development. Citrulline is primarily produced by small intestinal epithelial cells and is released into the bloodstream; however, the level of citrulline remarkably declines during intestinal injury. I-FABP is produced by intestinal epithelial cells and is released in the event of ischemia and injury of the intestinal villi. Research has indicated the potential clinical utility of the dynamic detection of citrullinated and I-FABP in monitoring the gastrointestinal function and integrity in septic patients (45). However, studies examining the methodology for functional stratification remain limited. Furthermore, the detection of citrulline and I-FABP binding proteins is also constrained by the limited availability of testing equipment in primary care settings. Thus, the identification of a rapid and cost-effective specific index for the assessment of intestinal system function remains elusive, indicating a potential avenue for future research.

### Immune system

An infection may initiate an immune response by the immune system against pathogenic microorganisms by promoting the release and secretion of cytokines or inflammatory mediators to maximize the removal of pathogenic microorganisms. However, it can result in the strong systemic inflammatory response syndrome, which is also called the “cytokine storm,” which can damage normal organ tissues, resulting in subsequent organ dysfunction or failure. Early initiation of standardized intensive therapy to suppress the early cytokine storm has demonstrated the potential to reverse the poor outcome of patients with severe pathogenic microbial infections (46). Accordingly, the integration of the immune system into the SOFA score is considered to better predict the morbidity and mortality associated with serious infections and septic shock.

Interleukin-6 (IL-6) is a soluble protein synthesized by T cells that induces B-cell development. The onset of the inflammatory response is accompanied by a rapid increase in the IL-6 level, which peaks within 2 h; this occurs well before the elevation of other biomarkers, such as the C-reactive protein and procalcitonin (PCT). It has been demonstrated to have a good diagnostic accuracy for a variety of diseases, such as COVID-19 (47). Furthermore, studies have indicated that IL-6 is a reliable indicator for predicting mortality in patients with sepsis in the ICU. The incorporation of IL-6 into the qSOFA score reportedly enhances the early prediction accuracy of multi-organ dysfunction (46).

Procalcitonin (PCT), a peptide precursor of the hormone calcitonin, is released into the bloodstream in response to severe systemic infections, particularly bacterial infections. PCT is frequently employed as a marker of clinical antimicrobial efficacy. Specifically, the cessation of antimicrobial therapy may be guided based on the measurement of PCT at a concentration of less than 0.5 mg/L, or below 80% of the peak value. PCT has been demonstrated to be an independent risk factor for sepsis. Furthermore, the combination of qSOFA and PCT may serve as an early diagnostic indicator of sepsis in the emergency department. Considering the aforementioned, incorporating IL-6 and PCT into the SOFA score for assessing the immune function is also apparently reasonable (46). Regardless, the threshold for the clinical application of IL-6 and PCT remains an ongoing debate, and urgent evidence for clarification in subsequent clinical studies is needed.

### Hormone (endocrine) system

The endocrine system is activated at an early stage in patients with sepsis, with inflammatory mediators and bacterial products leading to a decreased secretion of certain hormones, including vasopressin, insulin resistance, and hyperglycemia. In studies involving critically ill patients (children and adults), both elevated and depressed blood glucose levels have been reportedly linked to unfavorable outcomes. Notably, transient severe hypoglycemia during infectious shock in children has been identified as a risk factor for long-term adverse outcomes (44). However, the administration of insulin and other medications can effectively regulate elevated blood glucose levels within a specified range in clinical practice. Considering the aforementioned, blood glucose values and insulin use should be included in the new SOFA score as a measure of the hormone (endocrine) system. However, these findings were hypothetical, and sufficient clinical evidence is needed to ascertain the feasibility of this approach.

**Figure 1:**
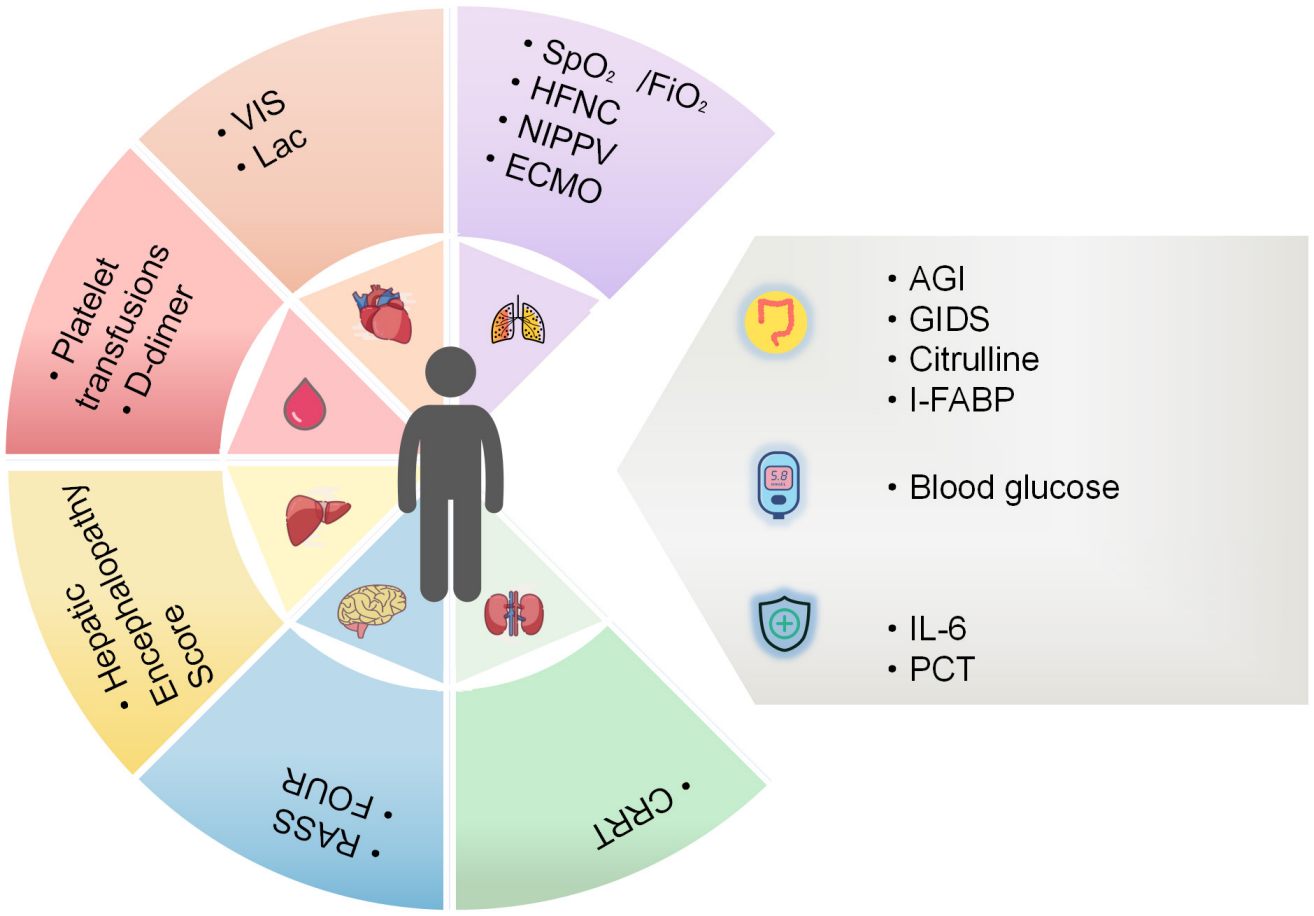
SOFA improvement strategies for current. The colored blocks list possible updates to the six organ systems in the SOFA score, and the gray blocks list possibly promising additions to the SOFA score. AGI, acute gastrointestinal injury; CRRT, continuous renal replacement therapy; ECMO, extracorporeal membrane oxygenation; FOUR, full outline of un-responsiveness; I-FABP, intestinal fatty acid binding protein; GIDS, gastrointestinal dysfunction score; HFNC, high-flow nasal cannula; NIPPV, non-invasive positive pressure ventilation; IL-6, Interleukin-6; PCT, procalcitonin; RASS; Richmond Agitation-Sedation Scale; SpO_2_/FiO_2_, peripheral capillary oxygen saturation/inhaled oxygen concentration; VIS, vasoactive inotropic score.

During the preliminary establishment phase of the SOFA-2 expert consensus, the gastrointestinal and immune systems were incorporated as new organ systems. Nonetheless, the internal validation phase, specifically the examination of the association between organ systems and mortality risk, revealed no significant correlation between gastrointestinal system scores and ICU mortality. The findings indicated a U-shaped relationship between immune system scores and mortality, suggesting that elevated and depressed white blood cell counts were associated with an increased risk of mortality. This observation precludes the establishment of a 0–4 point graded standard. Consequently, these systems are temporarily excluded. It is hoped that this review will provide a direction for future revisions (7). Beyond its clinical applications, SOFA demonstrates extensive utility in other domains. For instance, the utilization of longitudinal scoring data in conjunction with artificial intelligence technologies, such as machine learning models, facilitates the development of dynamic prognostic prediction models. This facilitates a closed-loop system encompassing “real-time assessment, risk alerting, and personalized treatment recommendations,” thereby enhancing the precision of critical care medicine. We have summarized the advantages and disadvantages of the new integrated organs/systems that could potentially be included in SOFA, as shown in Table 3, and mapped the SOFA improvement strategies (Figure 1).

**Table 3:** Advantages and disadvantages of the new integrated organs/systems.

**Organ/ system**	**Possible additions for consideration**	**Advantages**	**Disadvantages**
Intestinal system	• AGI or GIDS • Citrulline or I-FABP	• Non-invasive • Used widely • Specificity indicator	• Assessing complexity • incomplete • Invasive • Lake of stratification/ testing equipment
Immune system	• IL-6 • PCT	• High sensitivity • Good accuracy • Specificity indicator	• Invasive • Lack of stratification • Invasive • Lack of stratification
Hormone (endocrine) system	Blood glucose	Quick and easy testing	Affected by various factors

AGI, acute gastrointestinal injury; GIDS, gastrointestinal dysfunction score; I-FABP, intestinal fatty acid binding protein; IL-6, interleukin-6; PCT, procalcitonin.

## Conclusion

Considering the two-decade history of the SOFA score, we asserted the constraints of the six organ system scores and proposed potential solutions to these limitations. Additionally, we emphasized the necessity for the supplementary functional assessments of other systems, along with the possible indicators for their evaluation (Figure 1). In conclusion, an update to the SOFA score is necessary to align it with the evolving landscape of clinical practice and the growing diversity of patient needs.
