# Supplementary material for: Update of the sequential organ failure assessment score: current status and challenges?
Source: Front Med (Lausanne). 2026 Jan 13;12:1733090. doi: 10.3389/fmed.2025.1733090 (PMC12835212; doi:10.3389/fmed.2025.1733090)
Supplement: Supplementary file 1 [file Table_1.docx]

**Supplemental Table 1. Imputed PaO_2_ for a measured SpO_2_** ^[15]^

| Measured SpO**_2_** (%) | Imputed PaO**_2_** (mmHg) |
| --- | --- |
| 100* | 167** |
| 99* | 132* |
| 98* | 104* |
| 97* | 91* |
| 96 | 82 |
| 95 | 76 |
| 94 | 71 |
| 93 | 67 |
| 92 | 64 |
| 91 | 61 |
| 90 | 59 |
| 89 | 57 |
| 88 | 55 |
| 87 | 53 |
| 86 | 51 |
| 85 | 50 |
| 84 | 49 |
| 83 | 47 |
| 82 | 46 |
| 81 | 45 |
| 80 | 44 |
| 79 | 43 |
| 78 | 42 |
| 77 | 42 |
| 76 | 41 |
| 75 | 40 |
| 74 | 39 |
| 73 | 39 |
| 72 | 38 |
| 71 | 37 |
| 70 | 37 |

**Abbreviations:** *Generally considered unreliable on the basis of the sigmoidal shape of the hemoglobin-oxygen dissociation curve; **Based on SpO2 99.5%.
