# Supplementary material for: Update of the sequential organ failure assessment score: current status and challenges?
Source: Front Med (Lausanne). 2026 Jan 13;12:1733090. doi: 10.3389/fmed.2025.1733090 (PMC12835212; doi:10.3389/fmed.2025.1733090)
Supplement: Supplementary file 2 [file Table_2.docx]

**Supplemental Table 2.** **vasoactive inotropic score (VIS)** ^[26]^

| Drug | dose | factor |
| --- | --- | --- |
| Norepinephrine | μg/（kg-min） | 1 |
| Epinephrine | μg/（kg-min） | 1 |
| Dobutamine | μg/（kg-min） | 0.01 |
| Ddopamine | μg/（kg-min） | 0.01 |
| Milrinone | μg/（kg-min） | 0.1 |
| Levosimendan | μg/（kg-min） | 0.5 |
| Antidiuretic hormone | μg/（kg-min） | 100 |
