# Supplementary material for: Update of the sequential organ failure assessment score: current status and challenges?
Source: Front Med (Lausanne). 2026 Jan 13;12:1733090. doi: 10.3389/fmed.2025.1733090 (PMC12835212; doi:10.3389/fmed.2025.1733090)
Supplement: Supplementary file 3 [file Table_3.docx]

**Supplemental Table 3. Full Outline of Un-Responsiveness**

| Factor | Clinical manifestation | Score |
| --- | --- | --- |
| Eye response | Eyelids open or opened, tracking, or blinking to command | 4 |
|  | Eyelids open but not tracking | 3 |
|  | Eyelids closed but open to loud voice | 2 |
|  | Eyelids closed but open to pain | 1 |
|  | Eyelids remain closed with pain | 0 |
| Motor response | Thumbs-up, fist, or peace sign | 4 |
|  | Localizing to pain | 3 |
|  | Flexion response to pain | 2 |
|  | Extension response to pain | 1 |
|  | No response to pain or generalized myoclonus status | 0 |
| Brainstem reflexes | Pupil and corneal reflexes present | 4 |
|  | One pupil wide and fixed | 3 |
|  | Pupil OR corneal reflex absent | 2 |
|  | Pupil AND corneal reflexes absent | 1 |
|  | Absent pupil, corneal, and cough reflexes | 0 |
| Respiration pattern | Not intubated, regular breathing pattern | 4 |
|  | Not intubated, Cheyne-Stokes breathing pattern | 3 |
|  | Not intubated, irregular breathing | 2 |
|  | Breathes above ventilatory rate | 1 |
|  | Breathes at ventilator rate or apnea | 0 |
