# Supplementary material for: Update of the sequential organ failure assessment score: current status and challenges?
Source: Front Med (Lausanne). 2026 Jan 13;12:1733090. doi: 10.3389/fmed.2025.1733090 (PMC12835212; doi:10.3389/fmed.2025.1733090)
Supplement: Supplementary file 4 [file Table_4.docx]

**Supplemental Table 4. Gastrointestinal Dysfunction Score**

| 0- No risk  (No symptoms OR 1 of the following with oral intake) | 1- Increased risk  (2 of the following) | 1. GI dysfunction   (≥3 of score 1 OR ≥2 of the following) | 3-GI failure  (≥3 of the following） | 4- Life threatening  (≥1 of the following） |
| --- | --- | --- | --- | --- |
| 1. Absent bowel sounds  2. Vomiting  3. GRV >200 mL  4. GI paralysis/dynamic ileus  5. Abdominal distension  6. Diarrhea (not severe)  7. GI bleeding without transfusion  8. IAP >20 mmHg | 1. No oral intake  2. Absent bowel sounds  3. Vomiting  4. GRV >200 mL  5. GI paralysis/dynamic ileus  6. Abdominal distension  7. Diarrhea (not severe)  8. GI bleeding without transfusion  9. IAP >20 mmHg | 1. Severe diarrhea  2. GI bleeding with transfusion  3. IAP >20 mmHg | 1. Prokinetic use  2. GI paralysis/dynamic ileus  3. Abdominal distension  4. Severe diarrhea  5. GI bleeding with transfusion  6.IAP >20 mmHg | 1.GI bleeding leading to hemorrhagic shock  2.Mesenteric ischemia  3.Abdominal compartment syndrome |

**Abbreviations:** GRV, gastric residual volume; GI, gastrointestinal; IAP, intra-abdominal pressure.
